# Do relevant markers of cancer stem cells CD133 and Nestin indicate a poor prognosis in glioma patients? A systematic review and meta-analysis

**DOI:** 10.1186/s13046-015-0163-4

**Published:** 2015-05-14

**Authors:** Bin Wu, Caixing Sun, Fang Feng, Minghua Ge, Liang Xia

**Affiliations:** Zhejiang Cancer Hospital, 38 Guangji Road, Hangzhou, Zhejiang Province 310022 China

**Keywords:** CD133, Nestin, Prognosis, Glioma

## Abstract

**Background:**

CD133 and Nestin, as the markers of cancer stem cells, have recently been reported frequently in the pathogenesis and development of human gliomas. However, the prognostic role of CD133 and Nestin in gliomas still remains controversial. In this study, we aimed to evaluate the association between the expression of CD133 and Nestin and the outcome of glioma patients by conducting a systematic review and meta-analysis.

**Methods:**

We performed systematically electronic and manual searches through the database of Pubmed and embase (until to December 25, 2014) for titles and abstracts which investigated the relationships between CD133 and Nestin expression and outcome of glioma patients. A systematic review and meta-analysis was executed to generate Pooled hazard ratios (HRs) with 95 % confidence intervals (CIs) for overall survival (OS) and progression-free survival (PFS).

**Results:**

A total of 1,490 patients from 32 studies (13 articles) were included in the analysis. 19 studies and 13 studies investigated correlation between CD133 expression or Nestin and survival in gliomas, respectively. Our results showed that high CD133 expression in patients with glioma was associated with poor prognosis in terms of OS (HR 1.69; 95 % CI, 1.16–2.47; P =0.0060) and PFS (HR, 1.64; 95 % CI, 1.12–2.39; P = 0.010). In addition, high Nestin expression were associated with worse OS (HR 1.751; 95 % CI, 1.19–2.58, p = 0.004) but has no significant association with PFS (HR 1.55; 95 % CI, 0.96–2.51, p = 0.074). Even more important, the results of the subgroup meta-analyses show that that high CD133 expression was associated with worse prognosis in terms of OS and PFS in patients with WHO IV glioma but not WHO II-III. On the other hand, Nestin high expression was associated with worse prognosis in terms of OS and PFS in patients with WHO II-III glioma but not WHO IV.

**Conclusion:**

High level of CD133 expression trends to correlate with a worse OS and PFS in glioma patients, especially WHO IV gliomas and Nestin high expression trends to correlate with a worse OS in glioma patients especially WHO II–III, revealing both the markers of cancer stem cells may as the potential pathological prognostic markers for glioma patients.

## Introduction

Glioma is the most common primary brain tumor with the most grade malignancy, although in recent years the diagnosis and treatment of gliomas have made great progress, the prognosis of patients with glioma remains poor [1]. There is an urgent need to find a reliable marker to predict the prognosis of glioma, thereby providing the basis for the choice of a reasonable individualized treatment plan [2].

Cancer stem cell theory considers that the occurrence of tumors derives from some special cells, these cells are called cancer stem cells with the similar characteristics to embryonic stem cells, such as self-renewing and unlimited proliferation, multi-directional differentiation and anti-chemoradiotherapy and so on [3, 4]. Due to these characteristics of cancer stem cells, the traditional treatments, such as radiation and chemotherapy, can not effectively remove the cancer stem cells, the remaining tumor stem cells continue proliferation and differentiation, leading to tumor recurrence [4, 5]. There are a variety of markers used to isolate glioma stem cells, CD133 and Nestin are the most commonly used two markers that are widely expressed in various tumor cells, such as malignant glioma, liver cancer, ovarian cancer, colon cancer, lung cancer, etc. [6–11]. In recent years, a number of studies analyze the relationship between the markers of tumor stem cells CD133, Nestin and prognosis of patients with glioma, but due to differences in research method, sample size and the study population, the findings of a single sample are difficult to extend to the entire population and the obtained conclusions are inconsistent. This study used Meta-analysis method to systematically evaluate the literatures on the relationship between the expression of Nestin, CD133 that were multiple markers involving glioma stem cells and the prognosis of patients with glioma.

## Materials and methods

### Search strategy and study selection

A systematic literature search of the PubMed and Embase databases was conducted on studies evaluating the effect of the markers of cancer stem cells (CD133 and Nestin) on glioma patient survival. Our search strategy included terms (“Glioma” or “Glioblastoma”) and (“CD133 antigen” or “AC133 antigen” or “prominin-1” or “PROML1” or “Nestin”) and (“Survival” or “Mortality” or “Prognosis”). The literature search was conducted in 25 December 2014 and updated in 5 January 2015. Furthermore, a manual search of reference lists from the relevant original articles and review articles was also performed for additional relevant publications.

Two independent reviewers (Xia L and Wu B) independently inspected all candidate articles. Discrepancies were resolved by discussion. Studies that met all the following inclusion criteria were included in the review: (i) The diagnosis of glioma was made based on pathological examination; (ii) The association of the expression CD133 or Nestin with OS or PFS about gliomas was reported; (iii) The study provided the direct estimation of hazard ratios (HRs) and there was 95 % confidence intervals (CIs), or the date could be calculated by *p* values and other data reported. (iv) We included the studies with the largest sample size if the same glioma patient population were found to overlap among publications.

### Definitions and data extraction

The OS (overall survival) was defined as the time interval between the medical treatment and the death of patient or the last follow-up. The PFS (progression free survival) was calculated as the time interval between the date of treatment and the detection of the tumor recurrence or death from any cause. Both two reviewers independently carried out data extraction from including studies and any discrepancies were resolved by discussion between the two. The following data were extracted from all including studies: the first author’s name, year of publication, country, sample size, patient age, WHO grade, detect method of CD133 or Nestin expression, cut‑off level, follow up period, survival analysis and prognostic outcomes (PFS and OS). Any discrepancies were resolved through discussion amongst the authors.

### Quality assessment of primary studies

Quality assessment of included primary studies was independently executed by two reviewers (Xia L and Wu B) using the Newcastle–Ottawa Quality Assessment Scale (NOS). NOS scores of ≥6 were defined as high-quality studies. Any disagreement was determined by joint discussion.

### Statistical analysis

All analyses were performed by using stata 12.0 statistical software (Stata Corporation, College Station, TX, USA). Hazard ratio (HR) and 95 % confidence intervals (CI) were got directly from each study or from estimation of Kaplan-Meier survival curves according to the methods by Parmer *et al.* An HR less than one was defined as a better prognosis in glioma patients with IDH mutation, whereas an HR more than one indicated a poor prognosis. We the most powerful one (multivariate analysis was superior to univariate analysis. And the latter one weighted over unadjusted Kaplan-Meier analysis) was chose, if several HR estimates were presented in the same study.

The heterogeneity of the included trials was assessed by the Cochrane’s Q statistic for each meta-analysis. We carried out both fixed-effects (Mantel–Haenszel method) and random effects (DerSimonian–Laird method) models and producted the pooled HRs. Thanks to a priory of assumptions about the likelihood of heterogeneity across primary studies, the random-effects model was chosen. In addition, subgroup analyses were performed to investigate the potential causes of heterogeneity according to study country, sample size, patient age, follow-up period, detect method of CD133 or Nestin expression, cut‑off level and WHO grade.

Publication bias was first investigated by Funnel plots and then performed for each of the pooled study groups using the Begg’s test. All *p* values were two-sided and the significance level was set at 5 %.

## Results

### The study inclusion procedure and study characteristics

**Fig. 1:**
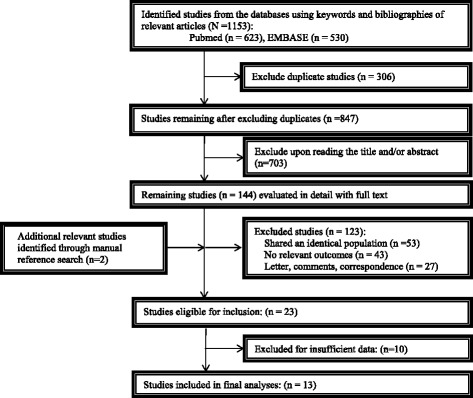
Flowchart of study selection

The selection procedure of the eligible studies was presented in Fig. 1. In brief, a total of 1153 studies were identified from our initial electronic search. Of these, we eliminated 306 studies owing to overlapping data sets. Of which, 847 abstracts were considered relevant and full texts were reviewed in detail. By the end of the review 13 literatures on glioma [12–24] (9 CD133 and 6 nestin; 1,490 patients), meeting our inclusion criteria for meta-analysis, were left with sufficient data for extraction.

The baseline characteristics of the literatures enrolled were summarized in Tables 1 and 2. Thirty two studies were included in those studies including 19 studies investigated the association between CD133 expression and outcome of glioma patients and 13 studies for Nestin. All studies were published between 2008 and 2014. Of these, the majority of the studies were executed in Europe (*n* = 23). Others were conducted in Asia (*n* = 8) and USA (*n* = 1). The total sample size from all studies was 1490 and the sample size was 24–379 patients and the range of medium age was 37.5–60.1 years. Of which, 13 studies evaluated grade II–III gliomas and 17 examined grade IV glioma. HRs and 95 % CI for OS or PFS in 27 studies could be directly extracted and was produced by Kaplan-Meier analysis for the 5 remaining studies. The most frequently used cutoff values for the high versus low/ present versus absent expression of CD133 or Nestin were the median (*n* = 15) and values calculated by using several semiquantitative methods.

**Table 1:** General characteristics of included studies (about the relationships between CD133 expression and OS or PFS in glioma patients)

First author	Year	Origin country	No. of patients	Median/mean age (year)	WHO grade	Detect method	Cut off level	Survival end points	Follow up period	Survival analysis	Adjusted variables
Ardebili [10]	2011	Slovenia	24	mean 60	IV	QRT-PCR	30 000 (─, 2-ΔΔCt > 30.000)	OS	NR	univariate (KM)	NR
Kase [11]	2013	Estonia	42	30–77	IV	IHC	median	OS	NR	multivariate	Radiotherapy dose, Chemotherapy, Karnofsky performance score
Kim [12]	2011	Korea	88	mean 54.9 (13–80)	IV	IHC	50 %	OS	mean 13.9 months (1–53)	multivariate	age, sex, Karnofsky performance scale (KPS) score, extent of removal, chemoradiotherapeutic modality, temozolomide modality, after temozolomide therapy and stem cell marker expression
								PFS			
Melguizo [13]	2012	Italy	75	24–81	IV	IHC	25 %	OS	NR	multivariate (KM)	NR
								PFS			
Metellus [14]	2011	France	48	mean 60.1 ± 9.2	IV	QPCR	1.03	OS	Median 18.9 months	multivariable	Age, extent of surgery, MGMT status (MethyLight)
								PFS			
Shibahara [15]	2013	Japan	112	Media 57 (7–77)	IV	WB	CD133/b-actin ratio = 1	OS	Median 25.7 months (3–152)	multivariable	Ki67 LI (≥35 %), 9p homozygous deletion and 10q loss
Shin [16]	2013	Korea	67	NR	IV	IHC	50 %	OS	NR	multivariable	status
								PFS			
Zeppernik [17]	2008	Germany	48	NR	II–III	IHC	1 %	OS	mean 86 months (±39)	multivariate	age, WHO grade and extent of resection
								PFS			
Dahlrot [18]	2014	Denmark	25	NR	II	IF	median	OS	Media 12 months	multivariate	age, performance status (PS) and IDH1 status
								PFS			
Dahlrot [18]	2014	Denmark	26	NR	III	IF	median	OS	Media 12 months	multivariate	age, performance status (PS) and IDH1 status
								PFS			
Dahlrot [18]	2014	Denmark	185	NR	IV	IF	median	OS	Media 12 months	multivariate	age, performance status (PS) and IDH1 status
								PFS

**Table 2:** General characteristics of included studies (about the relationships between Nestin expression and OS or PFS in glioma patients)

First author	Year	Origin country	No. of patients	Median/mean age (year)	WHO grade	Detect method	Cut off level	Survival end points	Follow up period	Survival analysis	Adjusted variables
Hatanpa [19]	2014	USA	50	median 37.5 years (20–66)	II–III	IHC	median	OS	4.3 year	multivariate (KM)	nestin, IDH, WHO grade, oligodendroglioma component (astrocytoma or oligoastrocytoma), MIB-1, age, sex, extent of resection and type of adjuvant, therapy
Milde [20]	2012	Russia	379	NR	II–III	IHC	50 %	OS	median 53 months	multivariate	cytogenetic group only, or cytogenetic group and posterior fossa group
								PFS			
wan [21]	2011	Germany	283	NR	II–IV	IHC	median	OS	Mean 11.21 ± 4.26 years	multivariate	WHO grade, patient age at diagnosis and extent of resection
Kim [12]	2011	Korea	88	mean 54.9 years (13–80)	IV	IHC	50 %	OS	mean 13.9 (1–53) months	multivariate	age, sex, KPS, extent of removal, chemoradiotherapeutic modality, temozolomide modality, after temozolomide therapy and stem cell marker expression
								PFS			
Arai [22]	2012	Japan	64	median 55 years (11–79)	III–IV	IHC	60 %	OS	1–67 months	KM	NR
Dahlrot [18]	2014	Denmark	25	NR	II	IF	median	OS	Media 12 months	multivariate	age, performance status (PS) and IDH1 status
								PFS			
Dahlrot [18]	2014	Denmark	26	NR	III	IF	median	OS	Media 12 months	multivariate	age, performance status (PS) and IDH1 status
								PFS			
Dahlrot [18]	2014	Denmark	185	NR	IV	IF	median	OS	Media 12 months	multivariate	age, performance status (PS) and IDH1 status

### CD133 expression and OS in gliomas

**Fig. 2:**
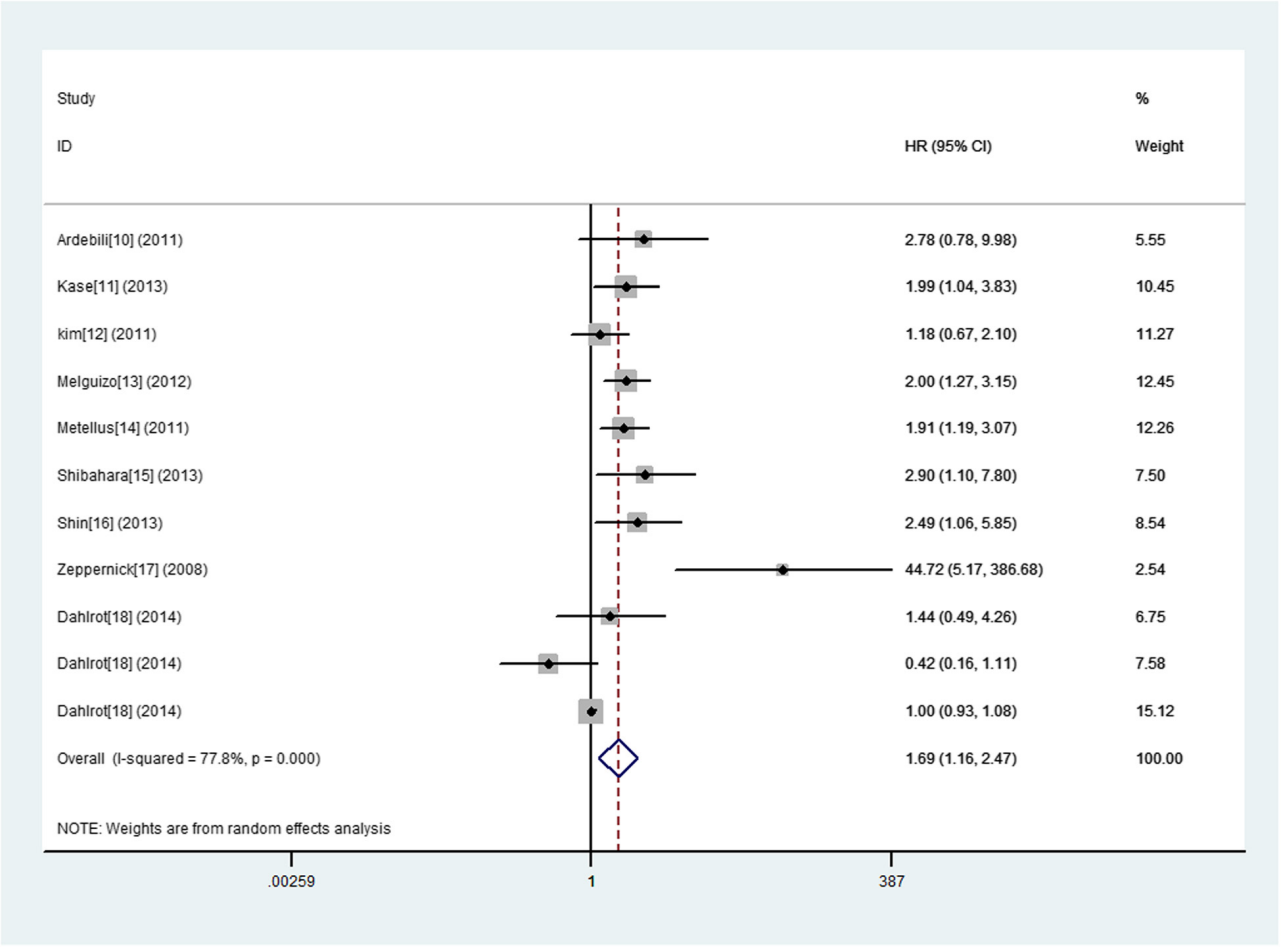
A forest plot of HR and 95 % CI of the association between CD133 expression and OS of gliomas

A total of 11 studies were involved in the association between CD133 expression and OS of glioma patients, among which statistically significant heterogeneity was observed (I^2^ = 77.8 %). Therefore, a random model was applicable to calculate a pooled HR and 95 % CI, the combined analysis showed that upon comparing patients with a low expression of CD133, patients possessing high CD133 expression had a significantly poorer OS (HR = 1.69, 95 % CI: 1.16 to 2.47; *P* = .0006)(Fig. 2).

In order to avoid the influence of heterogeneity, further subgroup analyses were conducted and stratified based on the study origin, sample size, follow up period, patient age, test method, cut‑off level and WHO grade. And the results showed that almost the subgroup analysis with origin country did not alter the prognostic role of glioma in OS substantially. And, increased expression of CD133 predicted a significantly worse OS in following subgroups including sample size ≤ 50, follow up period > media 12 months or no referred median/mean age referred, IHC test and cut‑off level not median. However, we did not discover any significant association in other subgroups (Table 3). Even more important, when in subtotal analysis stratified by WHO grade of glioma, 8 studies of WHO IV glioma exhibited a significant association between increased expression of CD133 and poor OS (HR 1.73; 95 % CI, 1.20–2.50, *p* = 0.004 I^2^ = 76.3). While, we did not find any significant association in subgroups of WHO II–III giomas (HR 2.38; 95 % CI, 0.30–18.82, *p* = 0.441 I^2^ = 87.0).

**Table 3:** Subgroup analyses of the relationships between CD133 ovexpression and overall survival or progression-free-survival

Comparison variables	Overall survival			Progression-free survival		
	Number of studies, Heterogeneity (I^2^ statistics; %)	HR 95 % CI, *P* value	Interaction, *P* value	Number of studies, Heterogeneity (I^2^ statistics; %)	HR 95 % CI, *P* value	Interaction, *P* value
Total	11 (77.8)	1.69 (1.16–2.47), 0.006	NA	8 (68.2)	1.64 (1.12–2.39), 0.010	NA
Origin country						
Europe	8 (81.0)	1.63 (1.03–2.61), 0.039	0.030	6 (76.5)	1.74 (1.06–2.83), 0.027	0.579
Asian	3 (42.5)	1.85 (1.02–3.35), 0.044		2 (0.0)	1.37 (0.86–2.18), 0.188	
Sample size						
>50	4 (77.4)	1.45 (0.91–2.31), 0.116	0.000	3 (0.0)	1.37 (1.01–1.73), 0.009	0.092
≤50	7 (67.6)	1.94 (1.07–3.54), 0.030		5 (77.0)	1.99 (0.94–4.22), 0.073	
Follow up period						
1,>media 12months	4 (74.1)	2.48 (1.13–5.45), 0.024	0.000	3 (86.7)	2.64 (0.98–7.11), 0.054	0.060
2,≤media 12months	3 (43.0)	0.90 (0.54–1.49), 0.678		3 (0.0)	1.12 (0.79–1.57), 0.530	
3,No Referreed	4 (0)	2.11 (1.52–2.93), 0.000		2 (0.0)	1.64 (1.19–2.27), 0.003	
Median/mean age y						
Referred	6 (0.0)	1.85 (1.45–2.37), 0.000	0.000	3 (0.0)	1.59 (1.24–2.04), 0.000	0.713
No Referred	5 (79.8)	1.57 (0.70–3.55), 0.274		5 (80.8)	1.79 (0.81–3.97), 0.153	
Test method						
IHC	5 (64.8)	2.16 (1.25–3.73), 0.006	0.000	4 (80.9)	2.25 (1.11–4.58), 0.025	0.122
Others	6 (71.2)	1.37 (0.85–2.22), 0.202		4 (20.4)	1.31 (0.93–1.84), 0.122	
Cut‑off level						
1:median	4 (61.4)	1.09 (0.66–1.79) 0.748	0.000	3 (0.0)	1.12 (0.79–1.57), 0.530	0.024
2:others	7 (51.3)	2.17 (1.44–3.27), 0.000		5 (74.5)	2.07 (1.25–3.43), 0.005	
WHO grade						
IV	8 (76.3)	1.73 (1.20–2.50), 0.004	0.915	5 (0.00)	1.46 (1.19–1.80), 0.000	0.143
II–III	3 (87.0)	2.38 (0.30–18.82), 0.411		3 (88.1)	2.17 (0.42–11.28), 0.355	

**Fig. 3:**
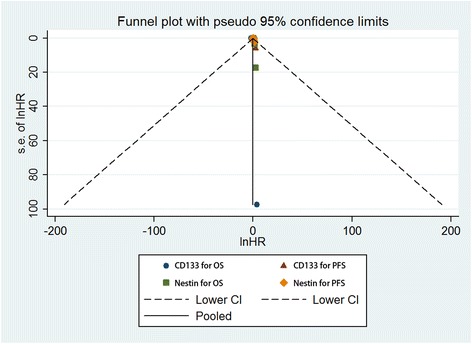
A Begg’s funnel plot for the publication bias test of the CD133 or Nestin overexpression and OS or PFS of human gliomas

Regarding the publication bias in the studies, we found no funnel plot asymmetry. Furthermore, Begger’s test was applied to provide statistical evidence for funnel plot symmetry. As expected, the *P* value of Begger’s test was 0.350 (Fig. 3). Hence, there was no evidence for significant publication bias in the meta-analysis.

### CD133 expression and PFS in gliomas

**Fig. 4:**
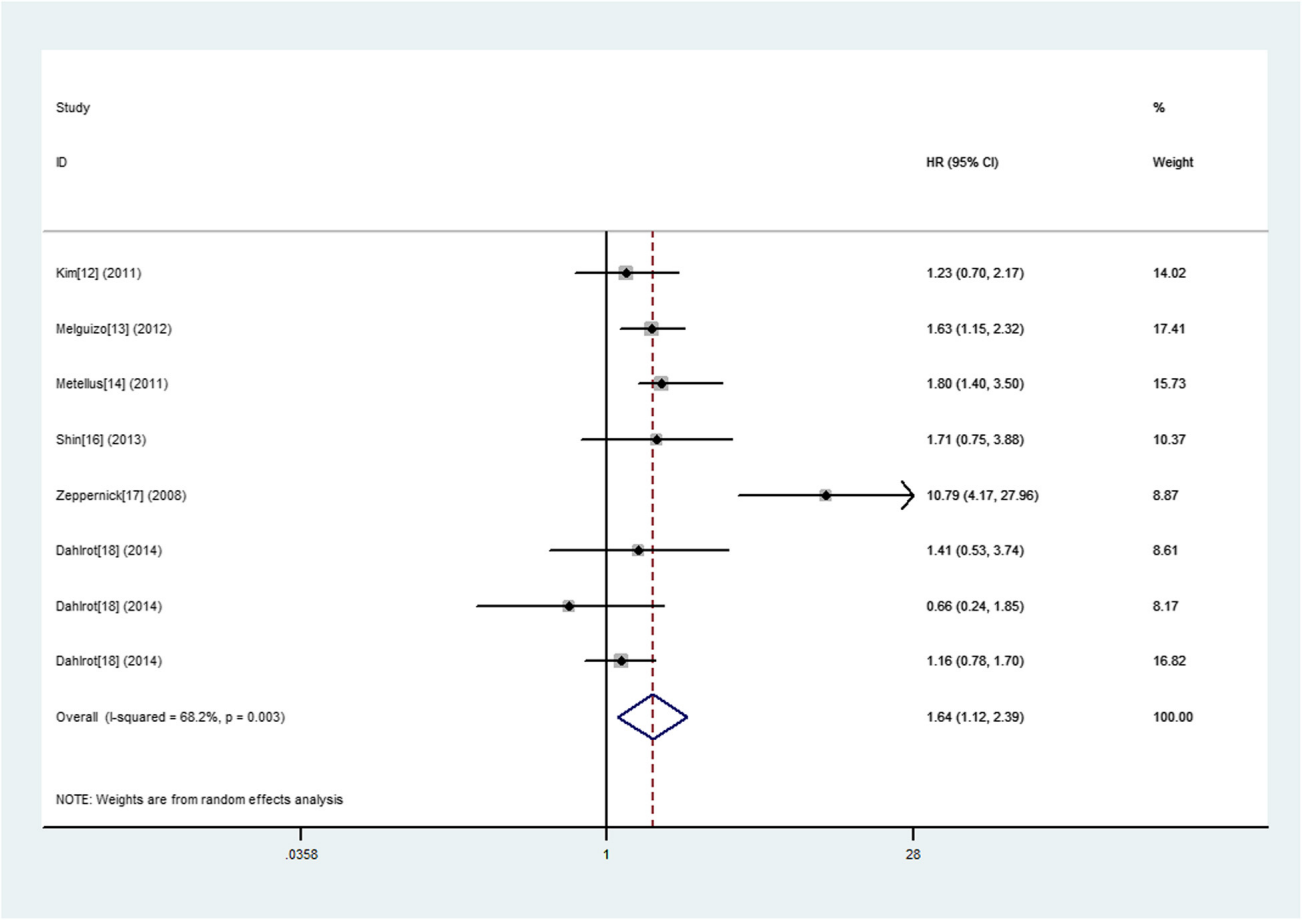
A forest plot of HR and 95 % CI of the association between CD133 expression and PFS of gliomas

Eight studies provided information concerning the association between CD133 expression and PFS of glioma patients. Similarly, a random model was applicable to calculate a pooled HR and 95 % CI, since significant heterogeneity was observed in the pooled studies (I^2^ = 80.5 %). The combined analysis exhibited a significant association between increased expression of CD133 and poor PFS (HR 1.73; 95 % CI, 1.86–2.83, *p* = 0.027) (Fig. 4).

Further subgroup analyses were conducted and stratified based on the study origin, sample size, follow up period, patient age, test method, cut‑off level and WHO grade. And the results show that increased expression of CD133 predicted a significantly worse OS in following subgroups including sample size ≤ 50, follow up period > media 12 months or no referred median/mean age referred, IHC test and cut‑off level not median. However, we did not discover any significant association in other subgroups (Table 3). Similarly, the results in subtotal analysis stratified by WHO grade of glioma showed that five studies of WHO IV glioma exhibited a significant association between increased expression of CD133 and poor OS (HR 1.46; 95 % CI, 1.19–1.80, *p* = 0.000 I^2^ = 0.00). However, we did not find any significant association in subgroups of WHO II–III giomas (HR 2.17; 95 % CI, 0.42–11.28, *p* = 0.355 I^2^ = 88.1).

At the same time, no funnel plot asymmetry was found in the studies and the Begger’s test did not show any evidence of publication bias (*P* = 0.902; Fig. 3).

### Nestin expression and OS in gliomas

**Fig. 5:**
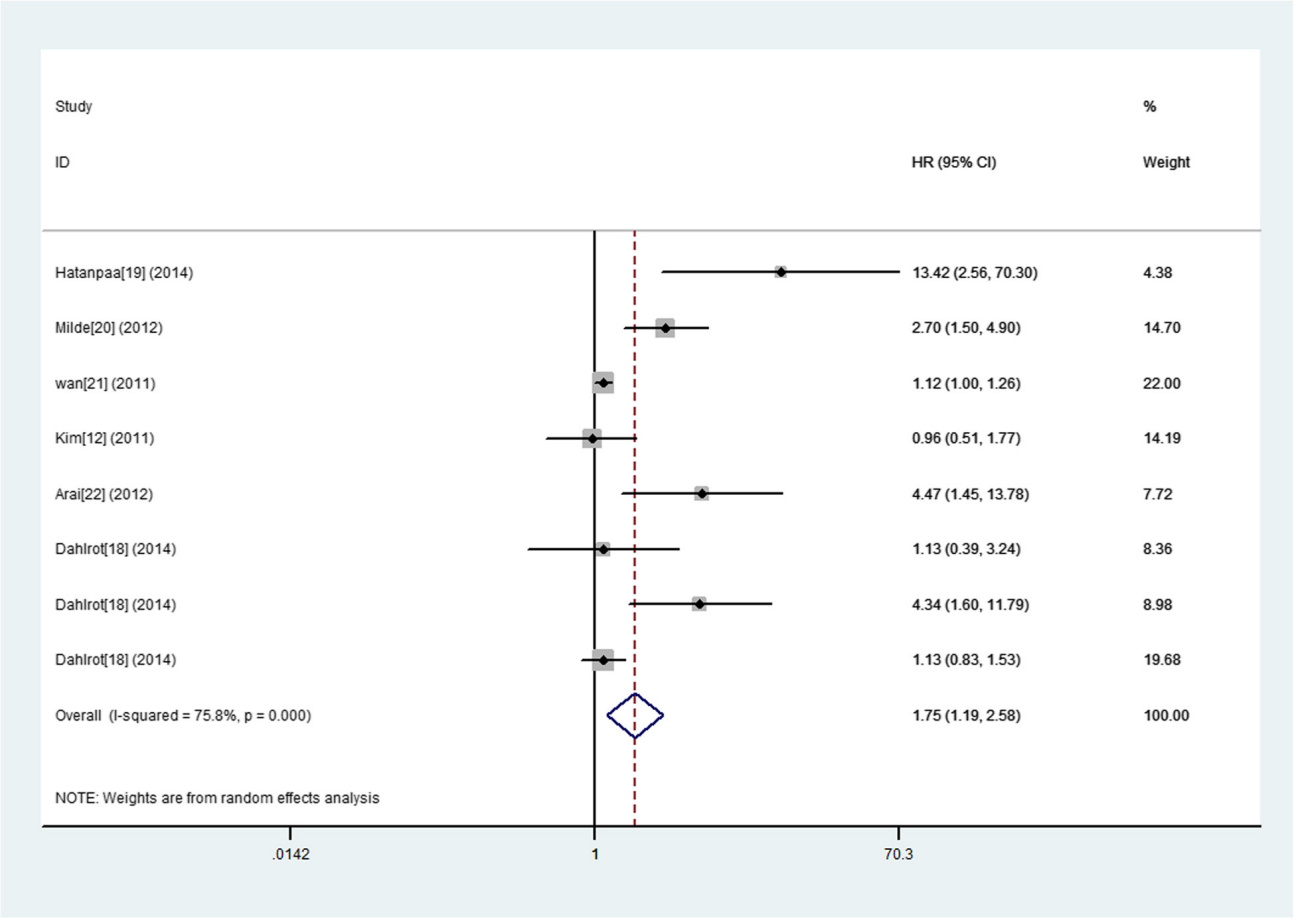
A forest plot of HR and 95 % CI of the association between Nestin expression and OS of gliomas

Eight eligible studies provided the estimation of the HR and 95 % CI for the correlation between Nestin expression and OS of glioma patients, among which statistically significant heterogeneity was observed (I^2^ = 75.8 %). Therefore, a random model was applicable to calculate a pooled HR and 95 % CI and the combined analysis showed that upon comparing patients with a low expression of Nestin, patients with high Nestin expression had a significantly poorer OS (HR = 1.75, 95 % CI: 1.19 to 2.58; *P* = 0.004) (Fig. 5).

The subgroup analyses were conducted and stratified based on the study origin, sample size, follow up period, patient age, test method, cut‑off level and WHO grade. And the results showed that increased expression of CD133 predicted a significantly worse OS in following subgroups including sample size ≤ 50, follow up period > media 12 months or no referred, median/mean age referred, IHC test and cut‑off level not median. However, we did not discover any significant association in other subgroups (Table 4). Even more important, when in subtotal analysis stratified by WHO grade of glioma, four studies of WHO II–III glioma exhibited a significant association between increased expression of Nestin and poor OS (HR 3.11; 95 % CI, 1.45–6.67, *p* = 0.004 I^2^ = 57.2). However, we did not discover any significant association in subgroups of WHO IV giomas (HR 1.09; 95 % CI, 0.83–1.44, *p* = 0.518 I^2^ = 0.000).

**Table 4:** Subgroup analyses of the relationships between Nestin expression and overall survival or progression-free-survival

Comparison variables	Overall survival			Progression-free survival		
	Number of studies, Heterogeneity (I^2^ statistics; %)	HR 95 % CI, *P* value	Interaction, *P* value	Number of studies, Heterogeneity (I^2^ statistics; %)	HR 95 % CI, *P* value	Interaction, *P* value
Total	8 (75.8)	1.75 (1.19–2.58), 0.004	NA	5 (71.5)	1.55 (0.96–2.51), 0.074	NA
Origin country						
Europe	5 (73.1)	1.52 (1.04–2.21), 0.029	0.014	4 (74.1)	1.76 (0.98–3.14), 0.057	0.142
Asian	2 (81.8)	1.92 (0.43–8.62), 0.393		1 (NA)	1.55 (0.96–2.51), 0.973	
USA	1 (NA)	13.42 (2.56–70.34), 0.002				
Sample size						
>50	5 (71.6)	1.41 (1.00–1.98), 0.051	0.005	3 (82.6)	1.39 (0.77–2.50), 0.277	0.322
≤50	3 (71.2)	3.61 (0.99–13.17), 0.052		2 (36.5)	2.14 (0.85–5.35), 0.105	
Follow up period						
1,>media 12months	2 (68.7)	4.96 (1.08–22.78), 0.040	0.001	1 (NA)	2.40 (1.60–3.40), 0.000	0.008
2,≤media 12months	3 (68.8)	1.64 (0.74–3.67), 0.225		3 (55.4)	1.49 (0.76–2.94), 0.246	
3,No Referreed	3 (66.8)	1.35 (0.77–2.38), 0.300		1 (NA)	1.01 (0.57–1.79), 0.973	
Median/mean age y						
Referred	3 (83.4)	3.36 (0.72–15.61), 0.123	0.154	1 (NA)	1.01 (0.57–1.80), 0.973	0.142
No Referred	5 (75.8)	1.52 (1.04–2.21), 0.029		4 (74.7)	1.76 (0.98–3.14), 0.057	
Test method						
IHC	5 (82.1)	2.11 (1.10–4.04), 0.025	0.671	2 (83.6)	1.60 (0.69–3.73), 0.275	0.063
Others	3 (68.8)	1.64 (0.74–3.67), 0.225		3 (55.4)	1.49 (0.76–2.51), 0.246	
Cut‑off level						
1:median	5 (74.1)	1.55 (1.00–2.42), 0.050	0.021	3 (55.4)	1.49 (0.76–2.94), 0.246	0.063
2:others	3 (75.6)	2.10 (0.88–5.00), 0.093		2 (83.6)	1.60 (0.69–3.73), 0.275	
WHO grade						
IV	2 (0.0)	1.09 (0.83–1.44), 0.518	0.000	2 (0.00)	1.04 (0.77–1.41), 0.808	0.000
II–III	4 (57.2)	3.11 (1.45–6.67), 0.004		3 (0.00)	2.34 (1.68–3.27), 0.000	

The publication bias in the studies was conducted, the funnel plot asymmetry was not found. Then, we applied Begger’s test to provide statistical evidence for funnel plot symmetry. As expected, the *P* value of Begger’s test was 0.266 (Fig. 3). Hence, there was no evidence for significant publication bias in the meta-analysis.

### Nestin expression and PFS in gliomas

**Fig. 6:**
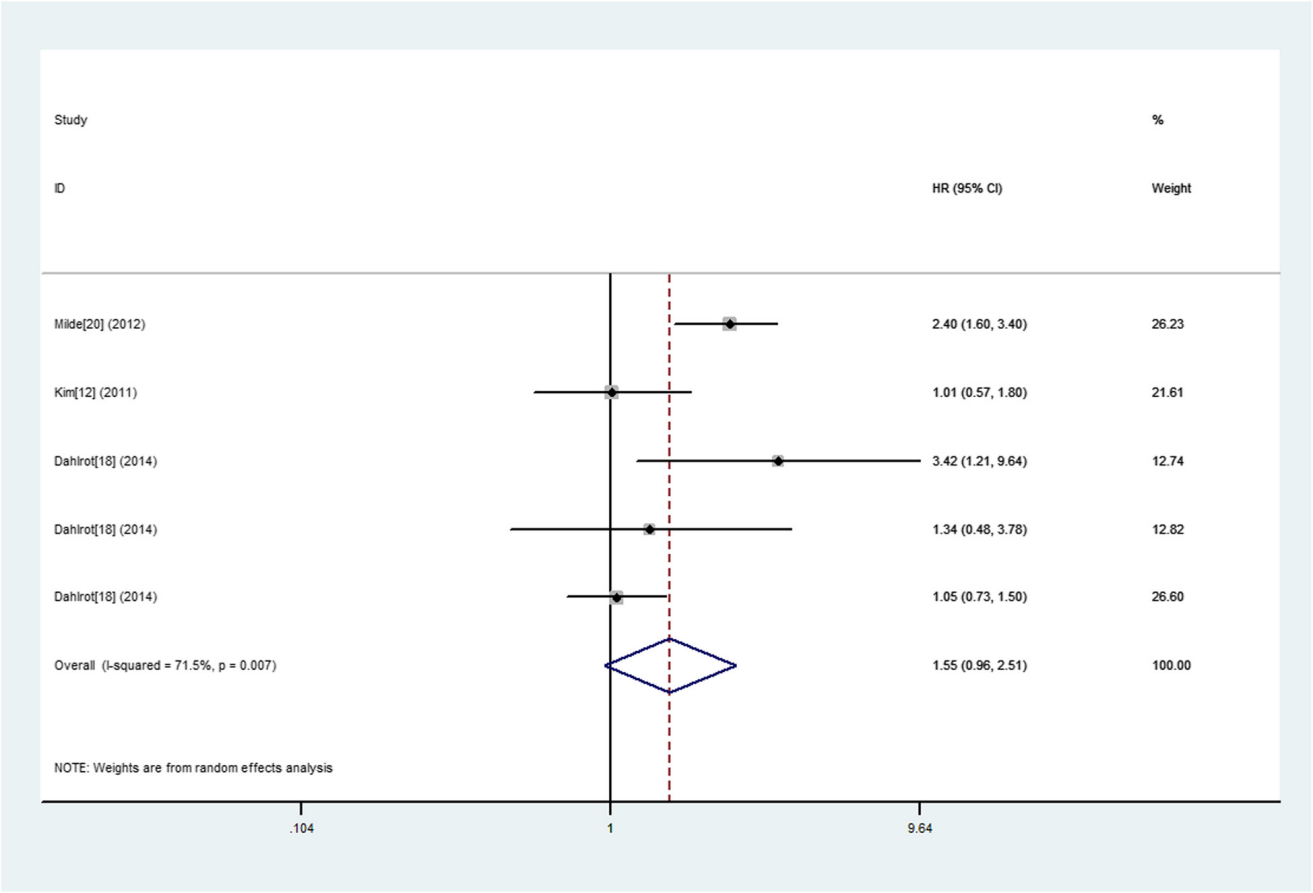
A forest plot of HR and 95 % CI of the association between Nestin expression and PFS of gliomas

The combined analysis of the five studies did not exhibit a significant association between increased expression of Nestin and poor PFS (HR 1.55; 95 % CI, 0.96–2.51, *p* = 0.074) (Fig. 6).

Further subgroup analyses were conducted and stratified based on the study origin, sample size, follow up period, patient age, test method, cut-off level and WHO grade. And the results showed that increased expression of Nestin predicted a significantly worse OS in following subgroups including sample size ≤ 50, follow up period > media 12 months or no referred, median/mean age referred, IHC test and cut‑off level not median. However, we did not discover any significant association in other subgroups (Table 4). Similarly, the results in subtotal analysis stratified by WHO grade of glioma showed that two studies of WHO IV glioma exhibited a significant association between increased expression of Nestin and poor OS (HR 2.34; 95 % CI, 1.68–3.27, *p* = 0.000 I^2^ = 0.000). However, we did not find any significant association in subgroups of WHO II–III giomas (HR 1.04; 95 % CI, 0.77-1.41, *p* = 0.808 I^2^ = 0.000).

At the same time, no funnel plot asymmetry was found in the studies and the Begger’s test did not show any evidence of publication bias (*P* = 0.221; Fig. 3).

## Discussion

Cancer stem cells (CSC), a small portion of cell population with the characteristics of stem cells, are present in the tumor tissue. It is the root to form the tumor cells with different degree of differentiation because of a capacity of self-renewing and the multi-directional differentiative potential [1, 3, 25]. At the earliest, CSC is found in the blood system tumors [26]. Recently, with the development of flow cytometry and in vitro tumor formation technology, cancer stem cells have been isolated and identified in a variety of solid tumors [27, 28]. The proposed cancer stem cell theory makes people have a new understanding on the biological behavior of tumors: the tumor is not only a genetic disease, but also a disease of stem cells. Stem cells become cancer stem cells after gene mutations, which is the root of tumor recurrence and metastasis [29].

Glioma stem cells can be sorted by finding its markers in order to conducted the targeted chemotherapy for glioma stem cells, which can effectively improve the specificity of chemotherapy and reduce the side effects on the normal engine body and cells, thereby finding a new breakthrough for cancer treatment [30]. Recently, the markers of glioma stem cell that are studied more than others include CD133, nestin, HMGA1, A2B5, etc. [31–34]. It has made some breakthroughs from the continuous in-depth study of these markers, but there is a lot of controversy.

At present, a number of studies have shown that glioma stem cell markers CD133 and Nestin are closely related to the prognosis of patients with glioma, but some individual studies show that there is no clear relationship between CD133, Nestin and the prognosis of patients with glioma. We used Meta-analysis to systematically evaluate the literatures on the relationship between the glioma stem cells markers Nestin, CD133 and the prognosis of patients with glioma in order to accurately and objectively evaluate the application value of CDl33 and Nestin in prognosis of glioma.

The current meta-analysis is the first to systematically estimate the association between cancer stem cell markers and glioma survival. In this study, the results showed that CSCs marker CD133 was associated with worse OS and PFS in glioma patients and Nestin was associated with worse OS but not PFS. Especially, subgroup analysis showed that the overexpression of CD133 had a more significant predictive value for glioma patients with WHO grade II–III, but Nestin for WHO grade IV.

Limitations of this study include: (1) the number of selected cases in the research is too few, especially only 1490 cases. (2) Although this study has tried to collect all the relevant data, the potential publication bias is inevitable, some data may be missing. The missing information may reduce the reliability of CDl33 and Nestin expression as a prognostic indicator of glioma. (3) Although immunohistochemistry and RT-PCR are the detection methods for CDl33, Nestin protein and gene, there are still some differences. This article collected all relevant documents in protein and gene level, there may be some heterogeneity in the methods. (4) the criterion of positive decision for the selected CDl33 and Nestin is different (median, mean, 25 %, 50 %, 1 %, or 3 others), leading to the heterogeneity of studies. (5) Follow-up time is different. So the study used a random effects model and subgroup analysis to compensate for these deficiencies.

Above all, we found that high CDl33 expression may be independent risk factor for glioma patients’ prognosis, especially WHO IV gliomas and high Nestin expression may be independent risk factor for glioma patients’ prognosis with grade WHO II–III. Based on the current findings, assessing CDl33 and Nestin expression could provide better prognostic information for patients with glioma and be used as a novel therapeutic target. Further large-scale cohort studies are needed to validate our results.
